# Leucocytic DNA Methylation of Interleukin-6 Promoter Reduction in Pre-Hypertensive Young Adults

**DOI:** 10.21315/mjms2019.26.6.5

**Published:** 2019-12-30

**Authors:** Wan Fatein Nabeila Wan Omar, Aszrin Abdullah, Norlelawati A Talib, Azarisman Shah Mohd Shah, Jamalludin Ab Rahman

**Affiliations:** 1 Department of Basic Medical Sciences, Kulliyyah of Medicine, International Islamic University Malaysia, Pahang, Malaysia; 2 Department of Pathology and Laboratory Medicine, Kulliyyah of Medicine, International Islamic University Malaysia, Pahang, Malaysia; 3 Department of Internal Medicine, Kulliyyah of Medicine, International Islamic University Malaysia, Pahang, Malaysia; 4 Department of Community Medicine, Kulliyyah of Medicine, International Islamic University Malaysia, Pahang, Malaysia

**Keywords:** epigenetics, pre-hypertension, pro-inflammatory, DNA methylations, young adults

## Abstract

**Background:**

Pre-hypertension is associated with increased risk of cardiovascular disease. Chronic inflammation plays an important role in the pathophysiology of essential hypertension, with epigenetic dysregulation involvement. Nevertheless, the role of DNA methylation in prehypertensive state is unknown. The aim of this study was to investigate the association between DNA methylation level of interleukin-6 (*IL-6*) promoter in pre-hypertensive (PreHT) and normotensive (NT) young adults.

**Methods:**

A total of 80 NT and 80 PreHT healthy subjects aged between 18–45 years were recruited in Kuantan, Pahang, Malaysia using an observational cross-sectional study approach. DNA methylation level of *IL-6* promoter in peripheral leukocytes were measured using bisulphite conversion and MethyLight assay.

**Results:**

There was no significant difference in age between NT and PreHT (*P* = 0.655). The mean blood pressure was 110(8)/73(5) mmHg in NT and 125(7)/82(5) mmHg in PreHT subjects. The *IL-6* promoter methylation level was significantly lower in PreHT compared to NT subjects (*P* < 0.001).

**Conclusion:**

The current study demonstrates that hypomethylation of *IL-6* promoter was associated with pre-hypertension in young adults. Thus, *IL-6* methylation could be used as an early indicator for predicting hypertension and related risk of cardiovascular diseases in prehypertensive subjects. Gene expression and longitudinal studies are warranted to examine the methylation effect on *IL-6* expression over time.

## Introduction

The 7th Report of the Joint National Committee on Prevention, Detection Evaluation and Treatment of High Blood Pressure described pre-hypertension as a condition with systolic blood pressure (SBP) of 120 mmHg to 139 mmHg and/or diastolic blood pressure (DBP) of 80 mmHg–89 mmHg (1). On that account, the aforementioned definition has been adopted in the Malaysia Clinical Practice Guidelines (CPG) on Management of Hypertension 4th edition (2). Recently, American Heart Association has reclassified pre-hypertension into ‘elevated’ and ‘stage 1 hypertension’ whereas the latest Malaysia CPG 5th edition re-categorised it to ‘normal’ and ‘at risk’ status (3, 4). Regardless of the nomenclature, strong evidence found that pre-hypertension as a major risk factor for hypertension and is associated with increased risk of cardiovascular diseases (5, 6). Globally, pre-hypertension is highly prevalent compared to hypertension, especially among young people with raised body mass index (7). In Malaysia, the prevalence of pre-hypertension in young adults is ranged from 10.5% to 50% (8–11). Although there is no academic consensus regarding the age of ‘young adults’, however, Levinson defined early adulthood as period between age 17 to 45 years (12).

The role of inflammation in essential hypertension has been well-established, either at systemic or localised vascular and renal level, by inducing oxidative stress and endothelial dysfunction (13, 14). Interleukin-6 (*IL-*6) is one of the most implicated pro-inflammatory cytokines associated with hypertension (13).

Epigenetics is a biological process that linking environment and genetic, and its dysregulation is involved in the pathophysiology of many complex diseases including essential hypertension (15). DNA methylation is an extensively explored epigenetic phenomenon, where its reversibility is promising in devising tools to aid prevention and therapeutic intervention in diseased state. Previous studies revealed that modification in *IL-6* methylation was seen in cardiometabolic diseases including essential hypertension *IL-6* (16, 17). Nonetheless, its correlation with prehypertensive state is unknown. The aim of this study was to investigate the association between DNA methylation level of *IL-6* promoter in prehypertensive (PreHT) and normotensive (NT) young adults.

## Subjects and Methods

### Study Design

The ethical approval for this study was obtained from Ministry of Health Malaysia (NMRR-16-2572-32869) and Institutional Ethic Committee (IREC 544). An observational cross-sectional study was conducted in Kuantan, Pahang, Malaysia from April 2017 to December 2017. A total of 80 PreHT and 80 NT subjects aged between 18 and 45 years were selected via purposive sampling based on the blood pressure readings obtained during health screening programmes. Malaysia CPG 4th edition was used for blood pressure classification, where PreHT was defined as subjects with SBP of 120 mmHg–139 mmHg and/or DBP of 80 mmHg–89 mmHg, whereas NT was defined as SBP of less than 120 mmHg and DBP of less than 80 mmHg. The exclusion criteria were as follows: participants with history of hypertension or on anti-hypertensive medications, previous diagnosis of chronic diseases including diabetes mellitus, ischemic heart disease, stroke, chronic renal failure, subjects on steroid medication or had hyper- or hypo-cortisolism and pregnant female participants or on hormonal contraception. Informed consent was obtained from all study participants, and the health status was confirmed through interview and self-administered questionnaire.

### Study Protocol

Study subjects were fasted for at least eight hours prior to sample collection that was conducted in Clinical Trial Unit at 0800 h. Blood pressure was measured using a recently calibrated automated blood pressure machine (Omron HEM-7130) and an appropriately sized cuff. Blood pressure was measured from the non-dominant arm positioned at the heart level whilst the subject was seated for at least 15 min. Three readings were taken with minimum 5 min interval in-between. Furthermore, 4 mL of venous blood was collected by a trained personnel into an ethylenediaminetetraacetic acid (EDTA) tube for methylation study. Sociodemographic data including sex, age, race, current smoking and alcohol habit and anthropometry measures (weight, height and waist circumference) were recorded. Participants with consistent high blood pressure reading (SBP ≥ 140 mmHg and/or DBP ≥ 90 mmHg) were referred to the primary healthcare team for subsequent management.

### Deoxyribonucleic acid (DNA) extraction and bisulphite conversion

Whole blood in the EDTA tube was centrifuged at 2000 g at room temperature for 10 min to obtain the leukocytes-rich buffy coat. DNA was extracted from the buffy coat using Gentra® Puregene® Blood Kit (Qiagen, USA) as per protocol described by the manufacturer. A total of 500 ng of DNA from each subject, Human Methylated & Non-methylated DNA Set (Zymo Research, USA) respectively was bisulphite-converted using EZ DNA Methylation-Gold Kit (Shallow-Well) (Zymo Research, USA) according to the manufacturer protocol and diluted to 10 ng/mL.

### MethyLight assay

The promoter sequences of *IL-6*, and *Alu* as the reference gene, were obtained from the Ensembl genome browser (www.ensembl.org). The forward and reverse primers, and probes were designed using the MethPrimer software tool (http://www.urogene.org/methprimer/) and procured as PrimeTime^®^ Std qPCR Assay (Integrated DNA Technologies, USA). Sequence of primers and probes used in the MethyLight assay were:

- *IL-6*: forward primer 5′-ATATTATATAGACG GATTATAGTGTACGGT-3′; reverse primer 5′-CGTAAACACTCCTAAACCAAATTCTCT-3′; and probe 5′-/56FAM/AAACGAAAC/ZEN/CACTACTCCCAACTCCGC/3IABkFQ/-3′.
- *Alu*: forward primer 5′-TGGTGATGGAGGA GGTTTAGTAAGT-3′; reverse primer 5′AACC AATAAAACCTACTCCTCCCTTAA-3′; and probe 5′-/56-FAM/ACCACCACC/ZEN/CAA CACACAATAACAAACACA/3IABkFQ/-3′.

The assay efficiency was determined prior to running the assay according to method described in a previous study (18).

For every sample, 25 ng bisulphite-converted DNA was added to 1x SensiFAST™ PROBE mix (Bioline, UK) and 1x PrimeTime^®^ Std qPCR Assay in a 10 mL polymerase chain reaction (PCR) using Bio-Rad CFX96™ Real-Time System (Bio-Rad, USA). *IL-6* and *Alu* were assayed in the same 96-wells plate. PCR reaction condition was 95 °C for 10 min (polymerase activation), followed by 95 °C for 10 s (denaturation) and 60 °C for 30 s (annealing and extension) and the cycle was repeated for 60 cycles. The *IL-6* methylation of each sample was assessed as $Cq\left[\frac{IL-6}{Alu}\right]=\frac{\left[CqIL=6\right]}{\left[CqAlu\right]}$.

### Data analysis

Statistical analyses were performed using IBM SPSS Statistics 22.0 software (IBM Corp, Armonk, New York). Categorical variables were described as number (percentage) and difference in proportion was analysed using Chi-square test. Normally distributed numerical variables were described as mean (standard deviation). The average SBP and DBP were calculated from the three measured blood pressure readings and mean arterial pressure (MAP) was calculated. Difference in mean was analysed using Student’s *t*-test. Bivariate association was performed using Pearson correlation test for normally distributed data. *P* < 0.05 was considered statistically significant.

## Results

### Sociodemographic Characteristics

Subjects age ranged between 18 and 45 years. There was no significant difference in age and race between NT and PreHT (Table 1). The proportion of male subjects was higher in PreHT group compared to NT group. There was no significant difference in proportion of current smoking and alcohol habit between the two groups. More than half of PreHT were obese, with a mean body mass index of 27.9 kg/m^2^. The waist circumference of PreHT males and females were both higher in contrast to NT (*P* = 0.010 and *P* = 0.019, respectively).

### Blood Pressure Parameters

The mean (standard deviation) of blood pressure measures among NT subjects was 110 (8)/73 (5) mmHg whereas PreHT was 125 (7)/82 (5) mmHg (Table 2). The MAP of NT group was lower by 8 mmHg compared to PreHT.

### IL-6 Promoter DNA Methylation

**Figure 1:**
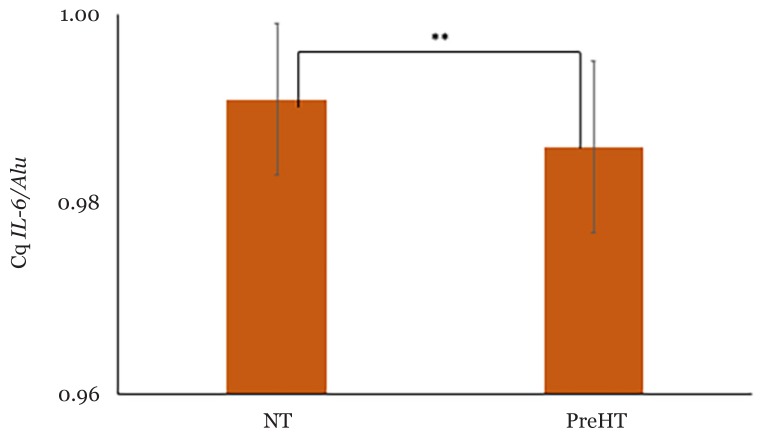
Mean DNA methylation level of *IL-6* promoter in young adults of different blood pressure category. Cq *IL-6/Alu* = DNA methylation level of *IL-6* promoter. NT (*n* = 80). Pre-HT (*n* = 80). Error bar represents standard deviation. Difference in mean is analysed using Student’s *t*-test. **P < 0.001

**Figure 2:**
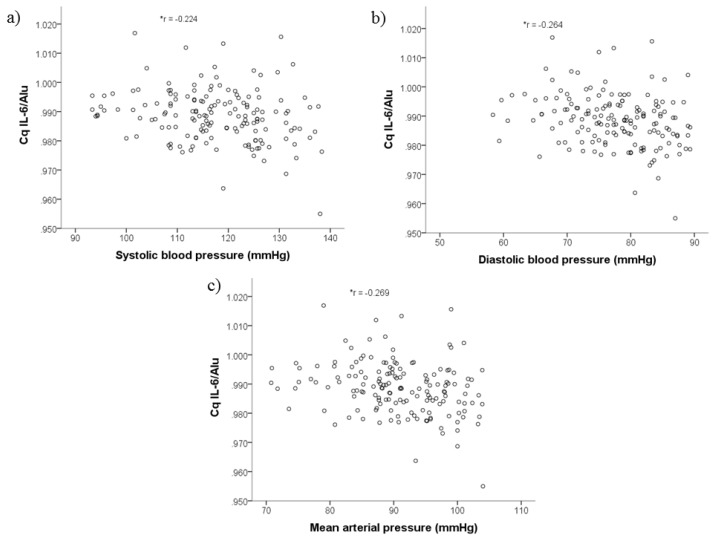
Association between DNA methylation level of *IL-6* promoter with blood pressure parameters: a) SBP; b) DBP; c) MAP. Analysed using Pearson’s correlation test. *P < 0.05, *r* = correlation coefficient

DNA methylation level of *IL-6* promoter was expressed as C_q_ *IL-6*/*Alu*. The mean (standard deviation) methylation level was significantly lower in PreHT [0.986 (0.009)] compared to NT [0.991 (0.008), *P* < 0.001] as shown in Figure 1. There was a significant inverse correlation between DNA methylation level of *IL-6* promoter and all blood pressure parameters (Figure 2).

## Discussion

This study was aimed to investigate the association between DNA methylation level of *IL-6* promoter in PreHT and NT young adults. The findings revealed that the methylation level was significantly lower in PreHT compared to NT, where it was inversely correlated with SBP, DBP and MAP.

DNA methylation level varies in response to the change in environment. Notably, methylation at promoter region could influence the gene expression at the transcription level, in which highly-methylated promoter region could suppress gene expression and vice versa.

The exact process relating to decreased methylation of the pro-inflammatory cytokine, *IL-6* to pre-hypertension is unknown. This could be due to a number of possible mechanisms that could explain this finding. First, low-methylated *IL-6* promoter region promotes *IL-6* expression, thus creating a systemic inflammatory environment, resulting in higher blood pressure. DNA methylation could repress gene expression by inhibiting the transcription factors from binding to the DNA promoter region during DNA transcription process either directly or indirectly via alteration of chromatin or histone structure (19, 20). Moreover, methylated cytosine is described to be less stable and is prone to mutation by converting to thymine (19). Also, DNA methylation could also regulate micro RNAs expression, which in turn can regulate other epigenetic regulators including DNA methyltransferase and histone deacetylase (21). Evidence has shown that CpG2 and CpG3 hypomethylation of *IL-6* was detected in hypertensive Chinese Han subjects, which might result in the upregulation of the inflammatory cytokine (17). Similarly, they reported significant inverse relationship between methylation level of both CpG sites with SBP and DBP (17). *IL–6* is an inflammatory mediator found abundantly in atherosclerotic plaque secreted by monocytes, endothelial cells and vascular smooth muscle (VSM) cells (22). *IL-6* is the chief trigger of hepatic cells to synthesise C-reactive protein (CRP), in which *IL-6* and CRP were both found to be at higher levels with increasing blood pressure (23–25). They act in concert to attract inflammatory cells to oxidised lipoproteins, eventually forming atherosclerotic plaque in the vascular tree (26). Secondly, *IL-6* impairs vascular relaxation by decreasing the bioavailability of nitric oxide, a potent vasodilator via various mechanisms; i) inhibiting endothelial nitric oxide synthase (eNOS) phosphorylation hence activation, and ii) prolonging caveolin–1 half–life, a negative eNOS regulator (27). Furthermore, *IL-6* could also stimulate hyperplasia of VSM cells by inducing platelet–derived growth factor synthesis (28). Abnormal proliferation of VSM cells contribute to atherogenesis. Interestingly, *IL-6* is also synthesised by VSM cells in response to Ang II as part of the atherogenic RAAS consequence. Altogether, *IL–6* impairs endothelial function and vascular relaxation resulting in increased arterial stiffness and peripheral resistance, hence causing higher blood pressure.

Secondly, the inflammatory environment from *IL-6* overexpression from hypomethylation induces obesity hence leads to higher blood pressure as part of obesity-associated metabolic syndrome. In this study, higher proportion of obesity and greater waist circumference were observed in PreHT than NT. As such, a previous study reported that *IL-6* and its receptor were overexpressed in the adipose tissue of obese subjects and positively correlated with body mass index (29). Obese people have higher mass of adipose tissue which is a chief ground of circulating *IL-6* (29). Obesity triggers a systemic oxidative stress (30), which is closely associated with pre-hypertension (31).

The third plausible explanation is the inflammatory state in obesity or higher blood pressure acts as the epigenator to trigger *IL-6* hypomethylation in preHT subjects. The role of *IL-6* as a pro-inflammatory and anti-inflammatory cytokine has been extensively discussed (32). Leukocytes are among the few cells, which expressed transmembrane *IL-6* receptors, thus *IL-6* could exert its anti-inflammatory action via the classical signalling mechanism (32). It should be noted that the metabolic inflammation might act as a negative feedback to promote anti-inflammatory activities of *IL-6* in order to suppress the inflammatory state.

To the best of our knowledge, this is the first report investigating the link between DNA methylation of *IL-6* promoter and pre-hypertension in young adults. *IL-6* hypomethylation holds a great potential in screening or early prediction of other hypertension-related diseases. The data could provide a valuable insight in understanding the molecular basis of pre-hypertension and could be extended to further in-depth molecular analysis.

### Limitations and Future Directions

Despite the significance of this study, the findings are limited due to the cross-sectional nature of the study. Thus, future research should include the longitudinal observational study approach in NT transitioning to PreHT or PreHT subjects converting to HPT, respectively. Alternatively, an experimental animal study should the effect of different level of *IL-6* methylation on the blood pressure. Also, the gene expression study should investigate the correlation between DNA methylation level and gene expression level in the future.

In this study, the DNA methylation of *IL-6* promoter was measured in the peripheral leukocytes as it was a minimally invasive method. There were discrepancies in the use of peripheral tissue to reflect the changes in the implicated organ. It should be noted that although leukocytes do not directly regulate blood pressure, the leukocytes circulate in the cardiovascular system and are closely related. Therefore, physiological changes in the leukocytes could affect the system to a certain degree. Ideally, measuring the DNA methylation level in the cardiovascular tissue such as cardiac tissue or blood vessel is preferable. The current study has attempted to minimise the white-coat effect by taking three blood pressure readings. Nevertheless, it could not be ascertained that the effect was completely eliminated.

## Conclusion

The current study indicates that hypomethylation of *IL-6* promoter was associated with PreHT in young adults. *IL-6* methylation could be an important indicator for predicting future hypertension in young adults. Gene expression and longitudinal methylation studies are warranted to establish the methylation effect on *IL-6* expression over time.

## Figures and Tables

**Table 1:** Sociodemographic characteristics of subjects

Sociodemographic aspect		Blood pressure status		*P-*value

		NT	PreHT	
Age (years)Φ		31 (7)	31 (7)	0.650^a^
Male*		40 (42.6)	54 (57.4)	0.025^b^
Race*	Malay	78 (51.7)	73 (48.3)	0.125^b^
	Others	2 (22.2)	7 (77.8)	
Smoking*		13 (38.2)	21 (61.8)	0.122^b^
Taking alcohol*		1 (33.3)	2 (66.7)	0.560^b^
Body mass index category*	Underweight/Normal (< 23 kg/m^2^)	31 (63.3)	18 (36.7)	< 0.001^b^
	Overweight (23–27.49 kg/m^2^)	28 (63.6)	16 (36.4)	
	Obese (≥ 27.5kg/m^2^)	21 (31.3)	46 (68.7)	
Body mass index (kg/m^2^)Φ		25.2 (5.6)	27.9 (5.7)	0.512^a^
Waist circumference (cm)Φ		84.9 (10.5)	92.1 (12.9)	0.140^b^
Female		83.4 (11.6)	90.4 (9.9)	0.019^b^
Male		86.4 (9.1)	93.3 (14.0)	0.010^b^

Notes:

Φ Mean (SD).

* *n* (row%).

a Analysed using Student’s *t*-test.

b Analysed using Chi-squared test.

NT = normotensive, PreHT = prehypertensive. Body mass index category is according to World Health Organization recommendation for Asian population

**Table 2:** Blood pressure parameters of each blood pressure group

Blood pressure parameters (mmHg)	NT	Pre-HT	*P*-value
SBP	110 (8)	125 (7)	< 0.001
DBP	73 (5)	82 (5)	< 0.001
MAP	85 (5)	97 (4)	< 0.001

Notes: Values expressed as mean (standard deviation). Difference in mean analysed using Student’s *t*-test
